# Unraveling the Complexity of the *N*_e_/*N*_c_ Ratio for Conservation of Large and Widespread Pelagic Fish Species: Current Status and Challenges

**DOI:** 10.1111/eva.70020

**Published:** 2024-10-10

**Authors:** Chrystelle Delord, Sophie Arnaud‐Haond, Agostino Leone, Jonathan Rolland, Natacha Nikolic

**Affiliations:** ^1^ UMR248 MARBEC, Univ. Montpellier Ifremer, IRD, CNRS La Réunion France; ^2^ UMR248 MARBEC, Univ. Montpellier Ifremer, IRD, CNRS Sète France; ^3^ Department of Earth and Marine Sciences (DiSTeM) University of Palermo Palermo Italy; ^4^ National Biodiversity Future Center Palermo Italy; ^5^ Centre de Recherche Sur la Biodiversité et l'Environnement (CRBE) Université de Toulouse, CNRS, IRD, Toulouse INP, Université Toulouse 3 – Paul Sabatier (UT3) Toulouse France; ^6^ Universite de Pau et des Pays de l’Adour, INRAE, AQUA, ECOBIOP Sain‐Pée‐sur‐Nivelle France; ^7^ ARBRE – Agence de Recherche Pour la Biodiversité à La Réunion Saint‐Gilles France

**Keywords:** conservation genetics, ecological genetics, population genetics – theoretical

## Abstract

Estimating and understanding the ratio between effective population size (*N*_e_) and census population size (*N*_c_) are pivotal in the conservation of large marine pelagic fish species, including bony fish such as tunas and cartilaginous fish such as sharks, given the challenges associated with obtaining accurate estimates of their abundance. The difficulties inherent in capturing and monitoring these species in vast and dynamic marine environments often make direct estimation of their population size challenging. By focusing on *N*_e_, it is conceivable in certain cases to approximate census size once the *N*_e_/*N*_c_ ratio is known, although this ratio can vary and does not always increase linearly, as it is influenced by various ecological and evolutionary factors. Thus, this ratio presents challenges and complexities in the context of pelagic species conservation. To delve deeper into these challenges, firstly, we recall the diverse types of effective population sizes, including contemporary and historical sizes, and their implications in conservation biology. Secondly, we outline current knowledge about the influence of life history traits on the *N*_e_/*N*_c_ ratio in the light of examples drawn from large and abundant pelagic fish species. Despite efforts to document an increasing number of marine species using recent technologies and statistical methods, establishing general rules to predict *N*_e_/*N*_c_ remains elusive, necessitating further research and investment. Finally, we recall statistical challenges in relating *N*_e_ and *N*_c_ emphasizing the necessity of aligning temporal and spatial scales. This last part discusses the roles of generation and reproductive cycle effective population sizes to predict genetic erosion and guiding management strategies. Collectively, these sections underscore the multifaceted nature of effective population size estimation, crucial for preserving genetic diversity and ensuring the long‐term viability of populations. By navigating statistical and theoretical complexities, and addressing methodological challenges, scientists should be able to advance our understanding of the *N*_e_/*N*_c_ ratio.

## 1 Introduction: Population Abundance and Effective Population Size in Conservation Genetics

As aptly enumerated by Swenson et al. (2024), abundance estimates and trends serve as essential metrics in conservation and resource management, playing key roles in assessing conservation status (Wilson, Kendall, and Possingham 2011), gauging the impacts of threats or recovery efforts (Jennings 2000; Ward‐Paige et al. 2012), and determining quotas (such as allowable biological catch and annual catch limits in fisheries) for managed populations of both target and non‐target species.

This paper focuses on large marine pelagic fish populations and the many logistical and conceptual challenges that researchers face when estimating their population abundance.

**FIGURE 1:**
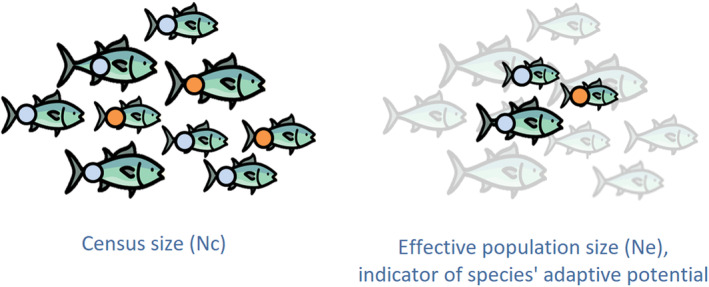
Schematic representation of the total abundance of a population on the left (census size—*N*_c_) and its effective population size (*N*_e_) on the right, with intrapopulation genetic variability depicted by blue and orange colored points in equal relative proportions. *N*_e_ reflects the size of an ideal, homogeneous population (on the right) that would experience the same level of genetic drift as the census‐size population (on the left). In scenarios of variable individual contributions to reproduction, the *N*_e_ of a population is generally lower than its total abundance *N*_c_. Here, genetic variation represented by the orange color is more likely to be eliminated from the population (genetic erosion) as it is less frequent in proportion compared to the genetic variation represented by the blue color.

The first set of challenges is logistic: marine pelagic fish are known for their extensive movements across the three dimensions of the ocean. Marine pelagic fish populations may be unevenly distributed due to environmental factors such as water temperature, salinity, reproductive period, seasonal migrations and prey availability, complicating the establishment of representative sampling methods. Abundance estimates are typically based on fisheries data, which are expected to reflect the values of fishery stocks and can be poorly documented. The ocean is an immense and often difficult‐to‐access environment, making comprehensive surveys of pelagic fish census population size (*N*_c_) costly in terms of time, resources, and logistics. Estimating the effective population size (*N*_e_) of marine pelagic fish populations can be an alternative or a complementary measure for several reasons. First, *N*_e_ estimation techniques may be less invasive than traditional fish capture and counting methods and can be estimated based on the modeling of genetic information that allows addressing a larger spatial scale with a lower number of individuals. This can be particularly important for threatened by‐catch: for example, scraping the skin of blue sharks with a pole when they are on the line and releasing them, as was done in a recent project (Nikolic et al. 2022). Second, *N*_e_ and its temporal fluctuations inform about a population's vulnerability to potential environmental disturbances and demographic trends, even for populations with high abundance such as large marine pelagic species (e.g., Waples et al. 2018). In fact, both *N*_e_ and *N*_c_ are pivotal factors in influencing the capacity of populations to withstand extinction risks arising from demographic, environmental, or genetic stochastic events (Palstra and Fraser 2012). These events encompass sporadic recruitment failures, inbreeding depression, or declines in genetic diversity when populations are small (Soulé 1987; Boyce 1992; Frankham et al. 2003 by Palstra and Fraser 2012). Consequently, they are of paramount importance to conservation geneticists (Leroy et al. 2017). *N*_e_ is not a direct measure of abundance, but rather a crucial metric in population genetics, reflecting the health and viability of a population. It can also relate to demographic factors affecting abundance. A small *N*_e_ reflects a substantial degree of genetic erosion experienced by a population, thus serving as an indicator of its adaptive potential (Figure 1). Between two populations with equal *N*_c_, the population with a smaller *N*_e_ is more likely to experience diminished genetic diversity, with a reduction of its adaptive potential compared to the one with a higher *N*_e_ (Ellegren and Galtier 2016).

For conservation purposes, it is important to distinguish the different types of population abundance and systematically provide a precise definition. First, various metrics have been employed to estimate the actual population size (*N*) also called the census population size (*N*_c_). *N*_c_ may also be named *N*_A_, *N*_B_, *N*_T_ (Frankham 1995), and *N*_a_ (Palstra and Fraser 2012). These metrics undercover slightly contrasted quantities, including juveniles and senescent individuals or only breeding adults for example. It is not always straightforward to navigate and provide accurate definitions for each metric, as publications often contain confusion or inexact correspondences. For instance, when examining the papers of Frankham (1995) and then subsequently those of Palstra and Fraser (2012), which are supposed to reiterate Frankham's definition of *N*_c_ in terms of *N*_A_, it becomes apparent that the definitions are not consistent. For example, Frankham (1995) uses the notation *N*_A_ to define abundance as the number of adults (*N*_A_ = breeding + senescent adults), whereas Palstra and Fraser (2012), citing Frankham (1995), define it as the annual census population size which is “The number of reproductively mature individuals in a population that may reproduce and hence contribute to the cohort of individuals born in that year” named in that publication *N*_a_ instead of *N*_A_. Waples (2024a) recommends using *N*_c_ as the number of adults alive at a given time to facilitate comparisons across species and emphasizes the importance of specifying whether juveniles are included or not. By synthesizing theoretical considerations and existing literature, we present a summary of various metrics in Table 1. Similarly, as conservation practitioners often focus on the number of reproducing adults (noted as *N*_B_ in Frankham (1995)), it is also important to avoid any confusion with the effective number of breeders *N*_b_, that we will mention in the next section, and which is more akin to *N*_e_. In population genetics, *N*_b_ is similar to a short‐term *N*_e_, scale of one reproductive cycle (instead of one generation), often used for specific periods such as a single breeding season for a given cohort (Waples and Teel 1990). The choice between the various *N*_c_ depends on the organisms being considered (Frankham 1995).

**TABLE 1:** Definition and notation of various metrics of census and effective population size used in literature and mentioned in the main text, with example studies.

Metrics	Definitions and notations	Example(s)	Outline of existing estimation methods
Census size *N*_x_	Total census size (*N*_T_): all individuals (juveniles + adults) in the population	Frankham (1995)	**Direct estimation** using classical approaches like counting, capture‐mark recapture (CMR), and telemetry **Indirect estimation using molecular markers**: e.g., close‐kin mark recapture (CKMR; Bravington, Skaug, and Anderson 2016), CRE (Creel–Rosenblatt Estimator; Creel and Rosenblatt 2013), the Moment estimator (Hettiarachchige and Huggins 2018), and the genetic‐based Capture–Mark–Recapture (g‐CMR; Müller, Mercker, and Brün 2020). For more details see also Larroque and Balkenhol (2023) **Deduction from the *N***_**e**_ **/*N***_**c**_ **ratio** if *N*_e_ is known
	Number of adults (*N*_A_): number of adult individuals (including both breeders and senescent) in the population	Frankham (1995), Waples (2024a)	
	Annual census population size (*N*_a_): number of sexually mature, i.e., potential breeder individuals contributing to the cohort of individuals born in that year	In Palstra and Fraser (2012), different from *N*_A_ in Frankham (1995)	
	Number of breeding adults (*N*_B_): number of sexually mature adults including sterile individuals and non‐breeding helpers	Frankham (1995)	
	Adult census size (*N*_c_): number of sexually mature, i.e., potential breeder individuals	Ferchaud et al. (2016), Waples et al. (2018)	
Effective size *N*_e_X	Variance effective size (*N*_e_ *V*): reflects contemporary effective size, especially the rate of changes in allele frequencies across generations		**Direct estimation using demographic information** when available, e.g., Hill (1972) for a diploid and dioecious species: $N_{e}=\frac{4N1*T}{\mathrm{Vk}+2}$ *N*1 being the cohort size at birth, *T* the generation time and *Vk* the lifetime variance in reproductive success across individuals from the cohort Many other equations were proposed to deal with various reproduction strategies (e.g., Wang, Santiago, and Caballero 2016), populations fluctuating in abundance (e.g., Engen, Lande, and Saether 2005) or structured populations (e.g., Wang and Caballero 1999; Gomez‐Uchida et al. 2013; Ryman, Laikre, and Hössjer 2019), some of them also requiring some genetic information **Deduction from the *N***_**e**_ **/*N***_**c**_ **ratio** if *N*_c_ is known and under certain conditions (e.g., the AgeNe method for a population of constant age‐structure and vital rates; Waples, Do, and Chopelet 2011) **Indirect estimation using molecular markers**, e.g., following the variation in allele frequencies between samples collected at several successive time periods (the temporal method, e.g., Jorde and Ryman 1995)
	Inbreeding effective size (*N*_e_I): reflects contemporary effective size, especially quantifying the rate of inbreeding (increase in homozygosity of alleles that are identical by descent)	Wright (1931) Crow (1954) is the first distinguishing *N*_e_I (originally defined by Wright) and *N*_e_V	**Deduction from the *N***_**e**_ **/*N***_**c**_ **ratio** if *N*_c_ is known and under certain conditions (e.g., the AgeNe method for a population of constant age‐structure and vital rates; Waples, Do, and Chopelet 2011) **Indirect estimation using molecular markers**: e.g., the sibship method (e.g., Wang 2009; Akita 2020a, 2020b)
	Linkage‐disequilibrium effective size (*N*_e_LD): generally used to estimate contemporary effective size using independent, unlinked molecular markers, but could also reflect effective size from more or less distant past time periods, depending on the physical linkage and the recombination rates between linked loci. When physical linkage among loci is know it can be used to reconstruct *N*_e_ trajectories	Waples, Larson, and Waples (2016) Santiago et al. (2020)	Sometimes considered analogous to *N*_e_V or *N*_e_I (Waples 2024a) but whose similarity to *N*_e_V and *N*_e_I actually remains unclear (Wang, Santiago, and Caballero 2016; Ryman, Laikre, and Hössjer 2019) **Indirect estimation using molecular markers,** as described by Hill (1981) and Waples (2006), with a recent review by Waples (2024b)
	Coalescent effective size (*N*_e_CO): typically reflects long‐term effective size and is associated with the overall genetic diversity (*θ*) of the focal populations	Kingman's coalescent (see also Sjödin et al. 2005 and Wakeley 2020)	The value θ (theta) is a composite parameter representing the product of the effective population size ($N_{e}$) and the mutation rate (*μ*). In population genetics, θ = 4 $N_{e}\mu$ is used to describe the genetic diversity and evolutionary history of a population, accounting for both the effective population size and the mutation rate **Indirect estimation using molecular markers** and the allele frequency spectra of one or more populations (e.g., Gutenkunst et al. 2009; Gattepaille, Günther, and Jakobsson 2016)
	Effective number of breeders (*N*_b_): reflects contemporary effective size per reproductive cycle, as opposed to generational effective population size	Crow and Denniston (1988) (see also Waples and Teel 1990; Waples, Do, and Chopelet 2011; Waples and Antao 2014)	**Direct estimation using demographic information** when available, e.g., *N*_b_ can be calculated for each sex (female—*f*, and male—*m*) (Crow and Denniston 1988): Nb=k¯N−2k¯−1+Vkk¯ and combined to obtain overall *N*_b_ per year (Wright 1938): $\frac{1}{N_{b}}=\frac{1}{4N_{\mathrm{bf}}}+\frac{1}{4N_{\mathrm{bm}}}$ With $V_{k}$ the lifetime variance in reproductive success and Vkk¯ the “index of variability” by Crow and Morton (1955) (Waples and Antao 2014). *N* is the number of individuals alive at any given time that have reached age at maturity. $N_{f}$ and $N_{m}$ is the number of adult females and males, respectively **Deduction from the *N***_**b**_ **/*N***_**c**_ **ratio** if *N*_c_ is known and under certain conditions (e.g., the AgeNe method for a population of constant age‐structure and vital rates; Waples, Do, and Chopelet 2011)
	Additive variance effective population size (*N*_eAV_): reflects contemporary effective size	Hössjer, Laikre, and Ryman (2016)	**Analytical approach** for diploid organisms

The second main challenge is conceptual and stems from the various definitions of *N*_e_ in the literature that have further contributed to confusion (Waples 2022). Initially, *N*_e_ is a genetic and evolutionary concept introduced by Sewall Wright in 1931 and 1938 as a theoretical number of individuals effectively contributing to the transmission of genetic diversity to the next generation, within an idealized population. It is defined in reference to the Wright‐Fisher idealized population (1922, 1930, 1931), a hypothetical finite population with simplified characteristics (detailed below) where genetic drift is the sole operative factor, and the dynamics of allelic and genotypic frequencies across generations depends solely on the population census size (Wang, Santiago, and Caballero 2016). Hence, the Wright‐Fisher model is commonly used to study the distribution of alleles in a population over time in the absence of evolutionary forces other than genetic drift. The idealized population under the Wright‐Fisher model should not be confused with that of Hardy–Weinberg, for the latter examines allele frequency equilibrium in infinite populations under otherwise similar idealized conditions as the ones from Wright‐Fisher. The Wright‐Fisher ideal population is more precisely described as an ideal population where each generation consists of a fixed number of individuals reproducing randomly, and each generation is the result of a random sampling of individuals from the previous generation (non‐overlapping generations), where changes in allele frequency are due to chance (genetic drift). So, each generation results from a binomial or multinomial (in the case of more than two alleles) sampling of gene copies from a pool of gametes, to which all individuals contribute equally. In such a population, the number of offspring produced by an individual follows a Poisson distribution. Since the 1960s (e.g., Kimura and Crow 1963), theories have emerged to predict the *N*_e_ under various inheritance modes and demographic scenarios. Subsequently, in the 1990s and 2000s (e.g., Schwartz, Tallmon, and Luikart 1999; Beaumont 2003), methodological advances have facilitated the estimation of the historical *N*_e_ of natural populations, owing to the rapid progress in molecular techniques and computational tools. Another manuscript dealing with this same special issue (Delord et al. 2024) is dedicated to these families of estimates. Considering these seminal definitions, it becomes evident that *N*_e_ cannot be simply equated to the notion of “Effective population size is the number of individuals that contribute offspring to the next generation.” Waples elucidates this concept exceptionally well in his 2022 publication. The number of parents contributing at least one offspring (*N*_p_) can be smaller or larger than *N*_e_. In practice, the *N*_p_/*N*_e_ ratio varies based on factors such as the sex ratio, the mean and variance in offspring number, and the relevance of inbreeding (Waples 2022). At this juncture, *N*_e_ can be defined as a concept in evolutionary genetics referring to a number of individuals and we will delve deeper into these distinctions in the forthcoming dedicated section.

In species with very large *N*_c_ such as marine fish (Hedrick 2005; Hedgecock and Pudovkin 2011), *N*_e_ often significantly lags behind *N*_c_ (*N*_e_ << *N*_c_, Frankham 1995). Studies of marine species have reported estimates of *N*_e_/*N*_c_ ratios ranging from 10^−3^ to 10^−8^ (Hauser and Carvalho 2008), implying *N*_e_ and *N*_c_ can, and apparently often, differ by several orders of magnitude.

For management and stock assessment purposes, it is essential to evaluate abundance trends to propose fishing quotas and ensure that ongoing exploitation is sustainable. Estimates based on fishing catch (e.g., catch‐per‐unit‐effort modeling and may concern the number of individuals or the weight (biomass)) and biological (e.g., age‐structured models) data are usually very difficult to collect and at best incomplete, hampering reliable *N*_c_ estimates. Alternative indirect methods for estimating abundance and demographic trends, such as tools based on the study of genetic variability, such as the *N*_e_ and *N*_e_/*N*_c_ ratio, are thus increasingly used to facilitate the estimate of *N*_c_. Moreover, the *N*_e_/*N*_c_ ratio is an indicator of the extent of genetic variation expected in a population (Hedrick 2005). *N*_e_, *N*_c_ and the *N*_e_/*N*_c_ ratio thus provide complementary information about the conservation status of a population (Hoban et al. 2020, 2024), yet their estimation also presents significant challenges. Those challenges may arise from specific methodological issues related to chosen estimation methods, comprehensively covered in detail by several reviews (Wang and Caballero 1999; Wang 2005; Wang, Santiago, and Caballero 2016; Waples 2016a) and analytical assessments (e.g., Waples, Larson, and Waples 2016; Gilbert and Whitlock 2015). In addition, the direct comparison of *N*_e_ and *N*_c_ of a population is not a trivial task and requires considerable caution to be biologically meaningful (Palstra and Fraser 2012). The purpose of this paper is to provide scientists and conservation practitioners with a comprehensive review of the critical issues affecting the assessment of a population's “abundance” (*N*_c_) and effective population size. We illustrate some of these issues with examples from large‐bodied, widely distributed marine species of high commercial or emblematic value with significant conservation concerns.

## 2 Different Types of Effective Population Sizes: Spatial, Temporal, and Evolutionary Consequences

In population genetics, the precise definition of *N*_e_ depends on the spatial and temporal scale considered, and there are actually several types of *N*_e_, all with specific implications in conservation biology. Firstly, it is crucial to determine the temporal scale for calculating *N*_e_. We typically distinguish contemporary *N*_e_ (“short‐term” or “contemporary effective population size”) from historical *N*_e_ (“long‐term” or “historical effective population size”), although these timeframes may partially overlap and form a continuum depending on the methods used (Nadachowska‐Brzyska, Konczal, and Babik 2022).

Short‐term *N*_e_ is relative to the present time or the last few generations of the sampled population. It reflects mostly the influence of the population's life cycle and demographic processes operating on recent time scales. It informs on the future prospects of a population and the risk of genetic erosion it may face in the near future (Wang, Santiago, and Caballero 2016). The most common contemporary *N*_e_ estimates used are those based on the loss of genetic diversity, specifically the loss of heterozygosity, through inbreeding (inbreeding *N*_e_) and genetic drift (variance *N*_e_) (Husemann et al. 2016). First distinguished by Crow (1954), they both refer to evolutionary processes occurring in one or a few recent generations (hence they are often referred to as “contemporary *N*_e_”) (Waples 2022). The variance *N*_e_ informs on the extent of genetic changes that can be observed within a given population over ecological time (Jorde and Ryman 1995, 2007). Even a small variance in *N*_e_ implies strong allelic frequency variations over time and thus a significant risk of genetic erosion (reduction in adaptive potential) through genetic drift.

Long‐term *N*_e_ generally focuses on the composite parameter *θ* = 4*N*_e_ *μ*, where *μ* represents the mutation rate (Watterson 1975 by Waples 2022). Long‐term *N*_e_ is relative to a population's evolutionary trajectory and reflects the combined influence of major evolutionary events, such as a bottleneck, founder effect or divergence events, occurring over a more or less ancient geological period (e.g., Pleistocene) on its current genetic diversity (Wang 2005).

Some methods (or combination of methods) can be used to estimate *N*_e_ at the present time, but also from various time periods in the past, hence capturing *N*_e_ trajectories through time (e.g., Santiago et al. 2020). In a conservation context, contemporary *N*_e_ is intuitively more meaningful, as it provides an expectation for future drift, rather than explaining how diversity got lost in the past. However, it is useful to compare it to an estimate of historical effective population size to replace the current health status of a population or stock in its evolutionary trajectory, and detect, for example, recent disturbances corresponding to increased anthropogenic pressures. Here, we can introduce the so‐called “coalescence effective population size” (often denoted N_eCO_), a long‐term effective population size related to the average number of generations for a genealogy of genes to converge to its most recent common ancestor.

For contemporary *N*_e_, we can introduce here the necessary distinction between the effective population size per generation or generational *N*_e_ and the effective population size per reproductive cycle (i.e., the effective number of breeders *N*_b_). They differ notably in the case of populations with overlapping generations (in contrast to an idealized theoretical population). Generational *N*_e_ informs on past and recent evolutionary processes influencing a population's genetic diversity and its future. *N*_b_, on the other hand, informs on the theoretical number of individuals contributing to the transmission of genetic information at a specific time step for a given cohort of individuals. It can thus be estimated for each reproductive cycle if information on several successive cohorts is available. *N*_b_ may be easier to estimate than generational effective population size and allows for understanding specific processes occurring at the scale of the reproductive cycle, such as sexual selection or density‐dependence (Waples and Antao 2014). Genetically based parentage methods are routinely used and are much easier to apply to a single season than to an entire adult lifetime (Waples and Antao 2014). The *N*_b_ parameter thus always represents a contemporary effective population size and can be estimated using the standard discrete‐generation formula for inbreeding effective size with separate sexes (Crow and Denniston 1988) in the software AGENE (Waples, Do, and Chopelet 2011; Waples and Antao 2014).

Contemporary *N*_e_ is typically linked to rates of change in allele frequency change or increase in inbreeding, while long‐term *N*_e_ is associated with nucleotide diversity (Waples 2022). In practical applications, it is probably more beneficial to define *N*_e_ using demographic parameters such as the number of potential parents (*N*), and the mean (*μ*_k_) and variance (σ_*k*_^2^) in offspring number per parent (*k*) (Waples 2022):

(1) InbreedingNe=μkN−1μk−1+σK2μk

(2) VarianceNe=μk2N−121+σK2μk

The “variance effective population size” (often denoted *N*_eV_) is generally contrasted with the “inbreeding effective population size” (often denoted *N*_eI_) (Table 1). While the former reflects the effects of genetic drift on the degree of allelic frequency variation over time, the latter reflects the increase in homozygosity that can occur in a population when the occurrence of mating between related individuals increases, following a decline in its abundance, for example, *N*_eV_ and *N*_eI_ may thus be identical or significantly differ within the same population. These values indeed reflect slightly different time periods, with *N*_eV_ describing an effective population size at the present time while *N*_eI_ describes the effective population size of the parental generation (Trask et al. 2017). Similarly, these values may differ if allelic frequency variations occur at a faster rate than mating between related individuals and the increase in the rate of homozygosity within the studied population.

Moreover, *N*_eV_ and *N*_eI_ will often differ in the case of structured populations (Wang and Caballero 1999), that is in any scenario other than the ideal case where all subpopulations are of equal size and exchange equal, constant, and symmetrical gene flow. In this last case, once migration‐drift equilibrium is reached, the *N*_eV_ and *N*_eI_ magnitudes at the overall metapopulation scale (Meta‐*N*_e_) are equivalent and converge towards a unique value (the so‐called “eigenvalue” effective population size, often denoted *N*_eig_, Ewens 1982; Wang and Caballero 1999; Ryman, Laikre, and Hössjer 2019). Furthermore, the sum of local *N*_eV_ yields this Meta‐*N*_e_ value, which then gradually converges towards *N*_eig_. At the local scale of each subpopulation, *N*_eV_ values can remain stable over time while *N*_eI_ values gradually approach the global Meta‐*N*_e_ value (Ryman, Laikre, and Hössjer 2019).

In nature, the classical scenario is that of subpopulations differing in abundance and connected by variable or asymmetric gene flow. The situation is thus most often more complex, necessitating not only a clear distinction between the relative scope of *N*_eV_ and *N*_eI_ but also between effective population size measures taken at the local (subpopulation) or global (Meta‐*N*_e_) scale, the latter no longer being calculable as a simple sum of local *N*_eV_ values (e.g., Gomez‐Uchida et al. 2013). Wang and Caballero (1999) and Hössjer et al. (2015) developed mathematical models predicting the local effective population sizes of each subpopulation in different situations (e.g., monoicous or dioecious populations, haploid or diploid, with equal or variable local abundances). The estimate of local effective population sizes generally involves demographic parameters such as local variance in reproductive success but also genetic parameters indicating the presence of gene flow between subpopulations, such as the fixation index *F*_ST_ (higher when gene flow between subpopulations is of low intensity). Hössjer, Laikre, and Ryman (2016) optimize some of these models for the specific case of diploid species and compare *N*_eV_ and *N*_eI_ magnitudes at the local and global scales (Meta‐*N*_e_).

Engen, Lande, and Saether (2005) propose a model predicting demographic effective population size (thus *N*_eV_) from the *N*_e_/*N*_c_ ratio in the case of fluctuating population sizes over time (expansion or decline). This model relies on Fisher's reproductive value concept (Fisher 1958) and on the assumed evolution of the frequency of a rare allele as a consequence of changes in age structure over time. Trask et al. (2017) apply this method to a population of Red‐billed Choughs, also integrating two *N*_eI_ effective population size estimation methods to compare them with Engen, Lande, and Saether's (2005) *N*_eV_ estimator. The latter can be calculated over a single generation or in the long term.

These different mathematical models that allow for direct estimation of effective population sizes are complex and rarely used on empirical datasets (but see Laikre et al. 2016; Trask et al. 2017), unlike indirect genetic estimation methods relying on linkage disequilibrium or allele frequency spectra. Nevertheless, they can be an interesting resource for the simulation of demographic scenarios and genomic data when the software eventually used to analyze or model data requires an approximate knowledge of the species. For example, when simulating a population structured by age, it is the *N*_c_ and demographic parameters (e.g., survival and fecundity rates) of each age class that can be set by the user (e.g., CKMRpop for forward‐in‐time simulation and tabulation of pairwise relationships in age‐structured populations (Anderson and Dunham 2005; Anderson 2023); PySLiM mentions the concept of *N*_e_ as an emergent property of an individual‐based simulation (see the link https://tskit.dev/pyslim/docs/latest/time_units.html?highlight=effective+size)). *N*_e_ can represent what can be called an “emergent property” of the simulated model. Its estimation will result from the demographic parameters set during simulation, which will notably influence the variance in reproductive success between simulated individuals, as well as from more stochastic variations related to how the chosen parameters are applied. For example, the method used to model genetic drift or the way random events are simulated can introduce variability in the *N*_e_ estimates. In such a case, it can be useful to use existing mathematical models first to (i) predict the theoretical *N*_e_ expected under a given simulation scenario deemed as realistic for the organism targeted, and then (ii) to estimate the *N*_e_ from data, to check their match. Moreover, if one wishes to subsequently test the performance of indirect estimation methods of *N*_e_ based on genetic data, it may be useful to estimate several theoretical and observed *N*_e_ values (e.g., values of local and global *N*_e_, short‐ or long‐term) to identify the scenario (and associated parameters) that best optimizes the convergence between theoretical or data‐based values.

From a more general perspective, it is essential to understand the different types of effective population sizes and the implications of their choice in theoretical and empirical contexts. An example provided by Hössjer, Laikre, and Ryman (2016) and taken up by Ryman, Laikre, and Hössjer (2019) is based on the concept of minimum viable population sizes (MVP, Shaffer 1981; Rai 2003; Reed et al. 2003) and specifically concerns the “50/500” rule also called Franklin‐Soulé number (Soulé 1980; Franklin 1980). This rule states a homogeneous and isolated population must have a minimum *N*_e_ of 50 in the short term (contemporary time scale, 1 to few generations) and a minimum of 500 in the long term (historical time scale) in order to maintain a stable population size through time. According to this rule, the smallest value best fits an inbreeding effective population size, *N*_eI_, and the largest an effective population size reflecting the loss of additive genetic variation (defined by Hössjer, Laikre, and Ryman 2016) denoted *N*_eAV_ (additive variance effective population size). The latter reflects the rate of loss of genetic variation due to the expression of phenotypic traits and is therefore directly related to the adaptive potential of an individual. Ryman, Laikre, and Hössjer (2019) show that different types of effective population sizes can vary greatly from each other within structured populations. They caution that some genetic tools available for estimating effective population size do not necessarily reflect *N*_eI_ or *N*_eAV_ and consequently cannot be used to verify the adequacy of an empirical population to the “50/500” rule. Moreover, the Franklin‐Soulé number was based on livestock populations that have been purged by humans, and may tolerate higher levels of inbreeding compared to wild species (Lande 1995, see also Clarke et al. 2024). Considering that only approximately 10% of the spontaneous mutational variance is quasi‐neutral (López and López‐Fanjul 1993a, 1993b), or for maintaining variability over 10 generations, it has been proposed that the Franklin‐Soulé number for wild species would need to be increased by a factor of 10, resulting in *N*_e_ = 5000 (Lande 1995).

## 3 Qualitative Prediction of the *N*_e_/*N*_c_ Ratio in Wild Populations: Influence of Life History and Demography

Understanding the influence of life history strategies and demography on *N*_e_ and *N*_e_/*N*_c_ is crucial for elucidating how these factors shape population resilience and the maintenance of genetic diversity. Variabilities in life history traits and demographic characteristics can significantly impact *N*_e_ and *N*_e_/*N*_c_, thereby influencing the long‐term viability of populations and their ability to adapt to environmental changes. Consequently, investigating this influence is essential for effective and sustainable management of wild populations. Significant work has been invested to identify general rules allowing estimating the *N*_e_/*N*_c_ ratio based on a population biology and life history traits (e.g., Hauser and Carvalho 2008; Waples et al. 2013; Wang, Santiago, and Caballero 2016; Waples 2024a). This, however, remains complex for wild populations (Palstra and Fraser 2012). On one hand, the reproductive strategies and life history traits of species influence their *N*_e_ by acting on the variance in reproductive success among individuals. For example, an imbalanced sex ratio can decrease the *N*_e_, as can any factor that generates non‐random mating among individuals (Nunney 1991, 1993, 1996), such as overlapping generations within age‐structured populations. Species with very high fecundity and a large number of offspring (albeit with a high mortality rate at young life stages), such as “r‐strategy” species—common among marine invertebrates—, often exhibit higher *N*_e_ and genetic diversity (Romiguier et al. 2014; Ellegren and Galtier 2016) than species with lower fecundity, producing fewer offspring (but more resistant, with lower mortality at young life stages), such as “K‐strategy” species. However, beyond these species' “typologies,” more demographic parameters will come into play. In iteroparous species (i.e., those that reproduce multiple times during their life such as large marine pelagic fish), *N*_e_ is influenced by life history traits such as longevity or age at maturity, which affect the variance in reproductive success (Lee, Engen, and Sæther 2011). Some of these life history traits may also influence the *N*_e_/*N*_c_ ratio among species. Waples et al. (2013) show that three demographic parameters influence this ratio predictably: (1) adult lifespan, (2) age at maturity, and (3) and the coefficient of variation in age‐specific fecundity. Indeed, a long adult longevity strengthens the influence of overlapping generations, reducing *N*_e_. Furthermore, a long‐lived species will have more numerous but also more spread out reproductive opportunities, with the risk that adult mortality between reproduction events period increases the variance in reproductive success among individuals and causes a decrease in *N*_e_. This phenomenon is even more pronounced if mortality rates are higher in younger and intermediate age classes than in older age classes (i.e., species with type III survival curves, Pinder, Wiener, and Smith 1978), or if fecundity increases with age. In this case, the greatest contribution to reproduction will essentially be ensured by the small proportion of old adult individuals that have survived to the most fecund age classes, again leading to increased overall variability in reproductive success among individuals and a decrease in *N*_e_. Fecundity parameters (including variations in fecundity among individuals of the same age, due to more or less favorable reproductive conditions, for example) as well as survival parameters, such as the annual adult mortality rate, thus have a significant influence on *N*_e_ and its relationship to total adult abundance (Waples 2016b). Barry, Broquet, and Gagnaire (2022) corroborate this finding through simulations, showing a pronounced decrease in *N*_e_/*N*_c_ as adult lifespan increases. This trend is particularly acute under type III survivorship curves when fecundity increases with age, a common trait among marine fishes. The authors also reveal an inversely proportional relationship between adult longevity (i.e., reduction in the expected *N*_e_/*N*_c_ ratio) and the observed genetic diversity in the genome of 16 species of marine fish in the Mediterranean Sea. The study of Barry, Broquet, and Gagnaire (2022) underlines the role of vital rates as critical drivers of *N*_e_ and genetic diversity levels within marine species in natural environments. In contrast, the rates of evolutionary processes are contingent upon *N*_e_ (Waples 2022).

Hill (1972) and Crow and Kimura (1970) mathematically summarize the relationship between *N*_e_ and the overall variance in reproductive success among individuals of the same cohort using the following equation, for a diploid species with separate sexes:

(3) $$N_{e}=\frac{4N_{1}*T}{V_{k}+2}$$

where *N*_1_ represents the total population size (or a cohort at birth), assumed constant over time, *T* represents generation time expressed as the average age of parents of a cohort at birth, and *V*_k_ represents the overall variance in reproductive success among individuals born in the same cohort.

Based on these theoretical and empirical findings, we could attempt to qualitatively predict the *N*_e_/*N*_c_ ratio of a species of interest if (i) we can accurately know its demographic parameters or assign it to a taxon or life history trait typology for which robust and consistent estimates are already available, and if (ii) we know the order of magnitude of one of the two values *N*_e_ or *N*_c_ in order to deduce the second. For example, Waples, Do, and Chopelet (2011) propose a method, implemented in the software AgeNe, allowing under certain assumptions the theoretical calculation of *N*_e_ and the *N*_e_/*N*_c_ ratio using demographic parameter values of a population. In practice, however, for many species, it is rare to know this information accurately. Furthermore, life history traits are not the only parameters likely to influence, in reality, *N*_e_ and the *N*_e_/*N*_c_ ratio (Waples 2024a).

Another complexity that remains unresolved but which will influence this ratio, as it has been demonstrated to impact *N*_e_, pertains to organism size. Recent research (Lynch et al. 2023) has revealed that *N*_e_ decreases with increasing organism size. This phenomenon may be correlated with the inverse scaling of mutation rates with genome size (Lynch et al. 2016) and the inverse relationship between recombination rates and genome size (Lynch et al. 2011). However, the relationship between body size and genome size is complex (e.g., Glazier 2021) and requires further investigation to understand the underlying mechanisms. These points introduce an additional layer of complexity to the determination of *N*_e_ values, which are crucial for accurately estimating a parameter that aids in deriving *N*_c_. Specifically, lower mutation rates in larger organisms could mean fewer genetic markers are available for estimating *N*_e_, reducing the precision of such estimates. Furthermore, decreased recombination rates could lead to larger blocks of linked genes, which can bias estimates of genetic diversity and, consequently *N*_e_. With lower recombination rates, linkage disequilibrium is higher because alleles within these larger blocks of linked genes are inherited together more often than by chance.

In marine species, for which very low *N*_e_/*N*_c_ ratios have been reported by numerous empirical studies (Hauser and Carvalho 2008), different life history strategies and typologies can be observed. Elasmobranchs, which include species of rays and sharks such as the blue shark, *Prionace glauca*, exhibit highly diversified reproductive strategies and migration behaviors that can diversely influence on *N*_e_ (Domingues, Hilsdorf, and Gadig 2018). The correlation between *N*_e_ and *N*_c_ in the sandbar shark, *Carcharhinus plumbeus*, the leopard shark, *Stegostoma fasciatum*, and the great white shark, *Carcharodon carcharias*, has suggested ratios close to 1 (*N*_e_ ~ *N*_c_) (Andreotti et al. 2016; Dudgeon and Ovenden 2015; Portnoy et al. 2009). These long‐lived species are characterized by a late maturity, a low fecundity remaining stable between age classes, and a low variance in reproductive success (Ovenden et al. 2016). However, other studies on species with similar life histories such as the blue shark (King et al. 2015) report lower *N*_e_/*N*_c_ values. Furthermore, the study of genetic diversity and *N*_e_ in elasmobranchs remains underexplored to date, with several scientific works highlighting the need for its better integration within conservation programs (Ovenden et al. 2016; Domingues, Hilsdorf, and Gadig 2018).

Species of large pelagic bony fishes such as tuna, swordfish, and sailfish, are likely to exhibit strong variations in reproductive success due to their high abundance and fecundity, which often increases with age. Furthermore, bony fishes often follow a type III survival curve with a high birth rate and high juvenile mortality, meaning that mortality rates are higher in young (and intermediate) age classes than in older age classes, with many individuals dying after few reproductive opportunities. Hedgecock (1994) proposed that exceedingly low *N*_e_/*N*_c_ ratios could result from a combination of prolific reproduction and elevated mortality among juveniles, leading to a significant variance in reproductive success substantially depressing *N*_e_ (Palstra and Ruzzante 2008). This concept has subsequently been completed and confirmed (Nunney 1996; Waples 2002; Hedrick 2005; Palstra and Ruzzante 2008). Thus, often in these species of type III, a large proportion of reproduction is ensured by a relatively small number of older and very fecund individuals. The sweepstakes reproductive success hypothesis (Hedgecock and Pudovkin 2011) also describes this phenomenon as one of the biological factors that may explain the very low *N*_e_/*N*_c_ ratios reported in these species (e.g., Laconcha et al. (2015) for albacore tuna, Laurent and Planes (2007) for sardines). Furthermore, sex‐ratio imbalances and temporal fluctuations in total abundance (linked, for example, to fishing exploitation) are also likely to influence the *N*_e_ and the *N*_e_/*N*_c_ ratio (Pinsky and Palumbi 2014). Nevertheless, the commonly high total abundance for such species theoretically implies *N*_e_ ranging from a few hundred to several thousand (or tens of thousands) individuals (Pinsky and Palumbi 2014). Thus, despite supposed low *N*_e_/*N*_c_ ratios, *N*_e_ itself can potentially be high, and its estimation can therefore be technically complex, due to challenges such as the need for large sample sizes (especially for highly abundant species) and potential bias from age structure. Waples (2016b) estimates that only extreme cases of sweepstakes reproductive success in marine teleosts could generate *N*_e_/*N*_c_ values actually lower than 10^−2^, a threshold well above the average of 10^−3^ to 10^−8^ reported above (see also Clarke et al. 2024). Moreover, many applications reporting low *N*_e_ and *N*_e_/*N*_c_ ratios would more likely reflect, in reality, the chosen method for *N*_e_ estimation than a true biological reality (Hare et al. 2011; Bierne, Bonhomme, and Arnaud‐Haond 2016; Waples 2016a; Irion et al. 2017). In southern bluefin tuna (*Thunnus maccoyii*), Waples et al. (2018) report a *N*_e_/*N*_c_ ratio on the order of 10^−1^, arguing that teleost life history traits do not systematically generate ratios as low as expected, despite the variability in reproductive success among individuals. Demographic parameters of teleosts may also vary between species. Thus, it cannot be excluded that even two species sharing relatively similar strategies could exhibit contrasted *N*_e_/*N*_c_ ratios if some of their demographic parameters differ (e.g., age at maturity, longevity, fecundity), as is the case with tunas (Juan‐Jordá et al. 2013; Murua et al. 2017).

It is therefore not easy to predict the order of magnitude of *N*_e_ and the *N*_e_/*N*_c_ ratio in large pelagic species based solely on broad categories of life history. In fact, although many estimates of the *N*_e_/*N*_c_ ratio between species were reported using both theoretical approaches (Waples et al. 2013) and reviews of empirical case studies (e.g., Frankham 1995; Palstra and Ruzzante 2008; Buffalo 2021), it remains difficult to derive general rules to date (Palstra and Fraser 2012). The respective estimation and linking of these two quantities therefore require greater investments and the development of dedicated scientific programs. Detailed knowledge of the demographic parameters of each stock could provide more insightful predictions, but such data are often lacking. Moreover, beyond the inherent biological characteristics of species, the influence of other demographic factors, such as migration patterns, reproductive behavior, and environmental variability, also warrants consideration. For example, fluctuations in total population abundance (resulting, for example, from fishing exploitation, temporal fluctuation in recruitment, and environmental variability) or the geographical scale considered (in the context of populations structured into several subgroups of various connectivity patterns according to a metapopulation model) influence both *N*_e_, and its relationship with *N*_c_. The *N*_e_/*N*_c_ ratio can also prove to be unstable over time depending on these factors (e.g., Baker et al. 2016). For example, fishing pressure exerted on a population can decrease *N*_c_ more rapidly than its *N*_e_, causing a temporary increase in the *N*_e_/*N*_c_ ratio (Allendorf et al. 2008). Repeated estimates over time are therefore necessary to better understand the potential vulnerability of a population or stock (Palstra and Fraser 2012). *N*_e_ and its relationship to *N*_c_ may also rely on the magnitude of *N*_c_ itself and some associated evolutionary parameters, such as recombination rates and linked selection, although consequent additional research should be carried on to better document these relationships (Ellegren and Galtier 2016; Buffalo 2021). Many other evolutionary factors like mutation, migration, and selection, as well as variations in recombination rate and linkage, can influence genetic variation (Waples 2022) and by consequence *N*_e_ and *N*_c_ (Waples 2024a). In populations with high abundance, the influence of selection can indeed outweigh that of genetic drift while in small population the random fluctuations in allele frequencies (genetic drift) mainly drives the fate of new mutations (Crow 1993; Lynch and Lande 1998).

In a general manner, documenting the *N*_e_/*N*_c_ ratio in species of conservation interest is recognized as an essential path of future research in conservation genetics. To better understand the influence of life history traits on the *N*_e_/*N*_c_ ratio, it is recommended to multiply estimations of both indices for various species and taxonomic groups (Hoban et al. 2024). Moreover, most methods for assessing population abundance (whether directly or indirectly using molecular markers) are relatively robust to low dispersal levels. In this respect, high cautiousness about statistical considerations and a good understanding of the spatial and temporal scope of *N*_e_ and *N*_c_ estimates will be essential and constitute the basis of the next sections of this summary.

## 4 Challenges in Relating *N*_e_ and *N*_c_

In this paper, we refrain from delving into specific statistical challenges and potential artifacts associated to each of the multiple methods to estimate *N*_e_ and/or *N*_c_. Those are widely available from a number of excellent reviews (e.g., Hauser and Carvalho 2008; Bravington, Skaug, and Anderson 2016; Wang 2016; Waples 2016a, 2024a, 2024b; Waples, Larson, and Waples 2016; Waples, Waples, and Ward 2022) and also constitute the basis of a detailed review in the present issue (Delord et al. 2024).

Beyond the biological challenges summarized above, exploring the intricate relationship between *N*_e_ and *N*_c_ is also associated to significant statistical challenges, often revealing complexities that extend beyond initial assessments (Palstra and Fraser 2012). It requires sometimes treating differently the generations or cohorts. Some indirect estimation methods (i.e., based on the study of genetic diversity rather than demographic parameters (Wang 2016; Wang, Santiago, and Caballero 2016)) of *N*_e_ provide information integrating several generations, while estimates of *N*_c_ are generally one shot and applied to the current time. Palstra and Fraser (2012) and Waples (2024a) provide an overview of good practices when aligning *N*_e_ and *N*_c_ across recent temporal scales or generations. Waples (2024a) indicates that “short‐term, single‐generation estimates of *N*_e_/*N*_c_ are the most meaningful and the best predictors of what to expect in the near future.” Waples et al. (2018) provide an example of cautious *N*_e_/*N*_c_ estimation on the Southern Bluefin tuna, and Waples (2005) an example of the effort to match contemporary *N*_e_ to the appropriate time periods when computing the *N*_e_/*N*_c_ ratio.

Relating *N*_e_ and *N*_c_ also implies being able to quantify correctly the degree of uncertainty around each of them, and the overall uncertainty associated with their ratio (Waples et al. 2018). However, the question of the best practices for constructing confidence intervals around estimates of *N*_e_ and *N*_c_ represents a field of research in itself (e.g., Hamilton, Tartakovsky, and Battocletti 2018; Waples, Larson, and Waples 2016; Jones, Ovenden, and Wang 2016). Finally, as mentioned in the Introduction, it is essential to clearly define and systematically report which *N*_c_ one aims to estimate. This quantity can indeed reflect the total population size, including immature individuals, or only adult and potentially reproductive individuals. The latter is recommended by Palstra and Fraser (2012) and by Waples (2024a), since it is the one that most directly influences *N*_e_.

Thus, although many estimates of the *N*_e_/*N*_c_ ratio between species are reported using both theoretical approaches (Waples et al. 2013) and reviews of empirical case studies (e.g., Frankham 1995; Palstra and Ruzzante 2008), deriving general rules for calculating the *N*_e_/*N*_c_ ratio remains challenging (Palstra and Fraser 2012) due to the complexity and variability inherent in the biological factors influencing *N*_e_ and *N*_c_ in different species and contexts. The respective estimation of these two quantities therefore often requires greater investments and the development of dedicated scientific programs.

To directly access *N*_c_ (in terms of number of breeders), alternative methods (Table 1) have emerged in recent years such as Close‐kin mark‐recapture (CKMR) (Bravington, Skaug, and Anderson 2016). This method exemplifies the requirements and challenges surrounding the development of *N*_c_ using genetic data. CKMR is a method based on the same principles as Capture‐mark‐recapture (CMR), where individuals are “marked” by their genotypes and considered “recaptured.” The number of parent–offspring (POP) relationships detected within a dataset, combined with the mathematical formulation of a demographic model based on vital population parameters (survival, fecundity, age at maturity, etc.), allows for the estimation of the most plausible total abundance (typically expressed as the number of breeding adult males and/or females). The CKMR model can generate robust estimates of population abundance when key assumptions such as annual reproduction and population size stability are met (Swenson et al. 2024). However, in cases of significant population declines or non‐annual reproductive dynamics, a more complex CKMR model must be constructed to avoid biased parameter estimates (Swenson et al. 2024). Despite the need for further improvements to address large marine pelagic fish, the recent CKMR literature demonstrates both the willingness and the necessity to develop tools for assessing the abundance trends and CKMR emerges as a promising tool for integration into long‐term monitoring programs (Swenson et al. 2024). Specific life‐history traits, such as semelparity and parthenogenesis, render CKMR methods inapplicable (Bravington, Grewe, and Davies 2016). The CKMR approach can provide crucial parameters for stock assessment (Rodríguez‐Rodríguez et al. 2022) and reduce uncertainty in these evaluations. For example, this method has been used to estimate the number of adult individuals of Southern bluefin tuna and to improve the definition of spawning stock biomass (Bravington, Grewe, and Davies 2014). Although we chose to detail CKMR in this paragraph due to its clear illustration of the principles and challenges of genetic *N*_c_ estimation, other methods such as CRE, the Moment estimator, and g‐CMR (Table 1) also offer complementary approaches for estimating population abundance. CMR methods based on POPs demonstrated significant potential for informing research and management strategies in species conservation, but their results must be interpreted with caution particularly considering the critical role of spatial and temporal sampling intensity (Larroque and Balkenhol 2023). Davies, Bravington, and Thomson (2017) demonstrated that CKMR population estimates for Atlantic bluefin tuna (*Thunnus thynnus*) can exhibit significant bias if the spatial structure is not explicitly considered. Another study revealed, through simulations, a positive relationship between population density and the sampling effort required for unbiased estimates (Rosenblatt et al. 2023). Finally, the design of a sampling strategy hinges on understanding the life history (demographic data) and behavior (spatial data) of the species studied (Rosenblatt et al. 2023).

## 5 Conclusion

In view of the challenges, even the impossibility, of determining individual numbers as is the case for large pelagic fish species, and the necessity to strive for minimally invasive technologies, molecular‐based metrics offer undeniable advantages. Recent advances and increasing interest aim to address the challenges surrounding tools using molecular markers. This summary highlights the critical importance of deepening our comprehension of *N*_e_ dynamics, particularly within the context of large pelagic fish species where the estimation of *N*_e_ and *N*_c_ is challenging and from which the estimated *N*_e_/*N*_c_ ratio is submitted to large variation within and among taxonomic groups.

Estimating the *N*_e_/*N*_c_ ratio is crucial for several reasons. Firstly, understanding *N*_e_/*N*_c_ aids in assessing the long‐term viability of species, especially in the face of environmental changes and anthropogenic threats. By doing so, it also provides valuable insights to inform conservation strategies aimed at preserving biodiversity, as accurate estimates of *N*_e_/*N*_c_ are essential for implementing effective management practices, such as stock management and habitat restoration, to ensure the persistence of wild populations.

Despite its importance, accurately estimating the *N*_e_/*N*_c_ ratio remains a challenge due to various technical and conceptual reasons. Technical challenges include the difficulty of gathering sufficient samples in large populations distributed across vast habitats, statistical limitations in estimating *N*_e_ and *N*_c_, particularly in species with complex life histories and spatial dynamics but also the characteristics of genomes. Moreover, uncertainties in demographic parameters and the inherent stochasticity of genetic processes further complicate accurate estimation. Fundamentally, the failure to estimate *N*_e_/*N*_c_ correctly stems from gaps in our understanding of the underlying biological mechanisms driving population dynamics, including factors such as reproductive strategies, migration patterns, and evolutionary processes.

To address these challenges, concerted efforts are needed to advance both theoretical frameworks and empirical methodologies for estimating *N*_e_ and *N*_c_. This requires interdisciplinary collaboration among geneticists, ecologists, statisticians, and conservation biologists to develop innovative approaches that account for the complexities of natural populations. Additionally, investing in long‐term monitoring programs and data collection efforts is crucial for improving the accuracy of *N*_e_/*N*_c_ estimates across diverse taxonomic groups and ecosystems. Furthermore, integrating advances in genomic technologies and computational methods can enhance our ability to capture the intricacies of population dynamics and refine estimates of the *N*_e_/*N*_c_ ratio.

In conclusion, by acknowledging the importance of estimating *N*_e_/*N*_c_, recognizing the challenges involved with long standing and new alternative methods, and proposing strategies for improvement, we can pave the road for more robust and reliable assessments of population health and genetic diversity. POPs‐based methods are promising alternative methods, but they are challenging as they necessitate extensive and prolonged sampling efforts. By addressing all the challenges head‐on, we can better inform conservation decisions and safeguard the future of wild populations and ecosystems.

## Conflicts of Interest

The authors declare no conflicts of interest.

## Data Availability

The authors have nothing to report.
